# Chronic Mesenteric Ischemia in a Patient With Limited Systemic Sclerosis

**DOI:** 10.7759/cureus.34209

**Published:** 2023-01-25

**Authors:** Ashley Barasa, Geoffrey Bader

**Affiliations:** 1 Internal Medicine, San Antonio Military Medical Center, San Antonio, USA; 2 Gastroenterology, San Antonio Military Medical Center, San Antonio, USA

**Keywords:** endovascular stent, vascular complications, gastroenterology, atherosclerosis, systemic sclerosis, chronic mesenteric ischemia

## Abstract

Chronic mesenteric ischemia typically develops secondary to the development of atherosclerosis within mesenteric vessels leading to the insufficient blood supply. While autoimmune conditions are an established independent risk factor for developing atherosclerotic plaques, the association between scleroderma and chronic mesenteric ischemia has been less studied. We present a case of a 64-year-old female with limited systemic sclerosis and atherosclerotic cardiovascular disease who presented to the Gastroenterology Clinic with progressive abdominal pain who was subsequently diagnosed with chronic mesenteric ischemia secondary to superior mesenteric artery stenosis and successfully treated with endovascular stenting.

## Introduction

Chronic mesenteric ischemia is a rare clinical entity defined as insufficient gastrointestinal blood supply to meet metabolic demands for at least three months [1]. Diagnosis requires a clinical history consistent with insufficient blood flow to the mesenteric organs, mesenteric artery stenosis of 70% or more in one or more vessels, and proof of mesenteric ischemia [2]. Classic symptoms of intestinal ischemia include postprandial epigastric abdominal pain, sitophobia, and weight loss [1].

Separate from the prevalence of mesenteric artery stenosis, the prevalence of symptomatic chronic mesenteric ischemia is relatively low due to extensive collateral circulation [2]. One prospective cohort study conducted by Kolkman and Geelkerken found an incidence rate of 5 to 6 per 100,000 and a prevalence of approximately 30 per 100,000, or 0.03% [2]. The likelihood of mesenteric artery stenosis progressing to chronic mesenteric ischemia increases with the number of vessels involved, with approximately 6% of those with multivessel mesenteric artery stenosis progressing to chronic or acute mesenteric ischemia within three to six years [2]. For those who develop symptomatic chronic mesenteric ischemia, 20% to 50% will progress to acute mesenteric ischemia [3].

Atherosclerosis leading to stenosis or occlusion of one or more mesenteric arteries is the primary cause of chronic mesenteric ischemia [1,4]. While traditional risk factors for atherosclerotic disease are well-recognized, there is a growing recognition that autoimmune diseases are an independent risk factor [5]. Systemic sclerosis is an autoimmune connective tissue disease of unknown etiology characterized by inflammation, vascular anomalies, and fibrosis, leading to the thickening and hardening of the skin and tissues [6-7]. These patients have been demonstrated to have an increased risk of atherosclerosis [5]. Here, we present an interesting case of a patient with limited systemic sclerosis with symptomatic superior mesenteric artery stenosis to highlight this association.

## Case presentation

A 64-year-old female with a history of limited systemic sclerosis, hypertension, hyperlipidemia, and coronary artery disease complicated by a myocardial infarction requiring percutaneous coronary intervention three months prior presented to the Gastroenterology Clinic for evaluation of abdominal pain. The patient was diagnosed with limited systemic sclerosis after meeting calcinosis, Raynaud's phenomenon, esophageal dysmotility, sclerodactyly, and telangiectasia (CREST) syndrome criteria at age 54 years and is currently managed with sildenafil alone for Raynaud's symptoms after previously receiving treatment with several immunosuppressants. The patient was diagnosed with coronary artery disease at the age of 56 years by left heart catheterization, and at present, due to her diffuse multivessel disease, she remains on dual antiplatelet therapy until she can receive further revascularization therapy.

**Figure 1:**
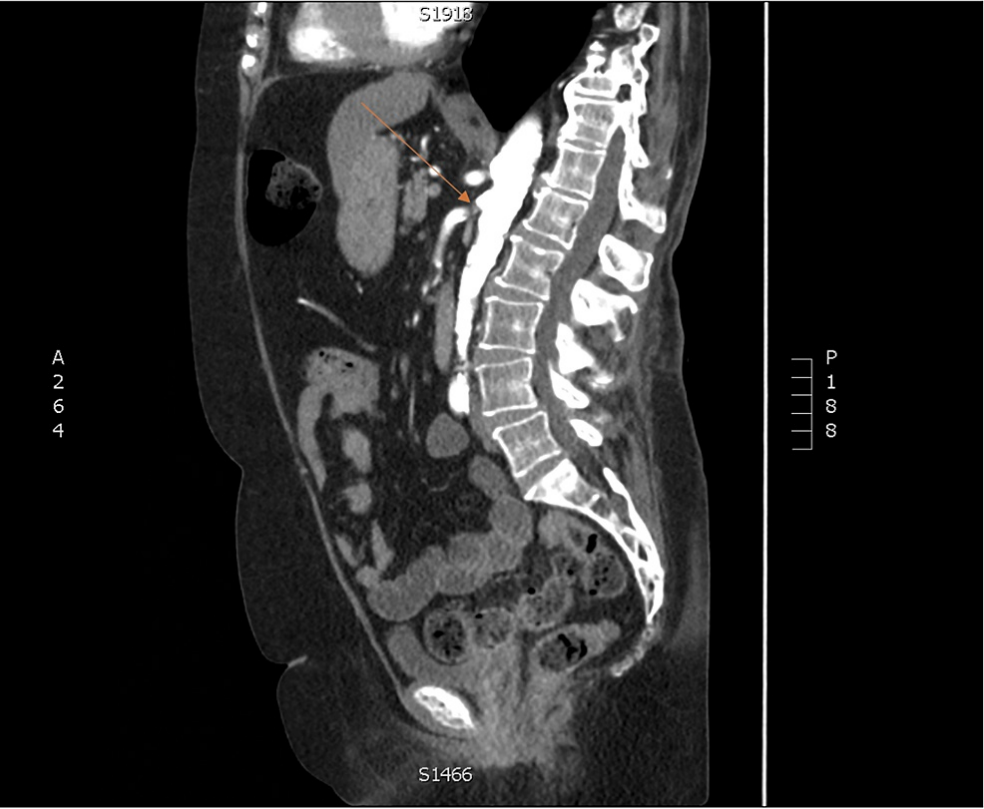
Sagittal CT angiogram of the abdomen and pelvis of the patient demonstrating the stenosis in the superior mesenteric artery (arrow). CT, computed tomography

**Figure 2:**
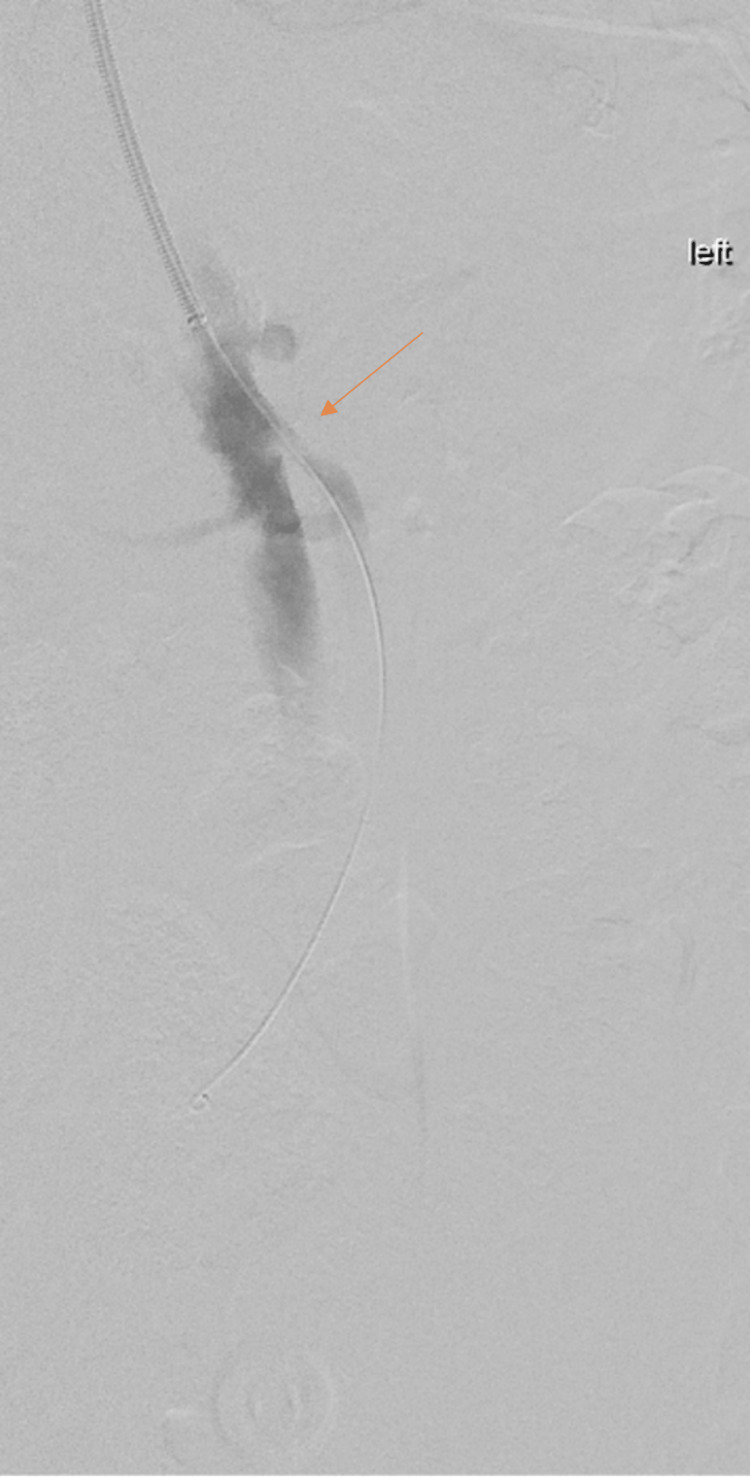
Mesenteric angiogram demonstrating stenosis of the superior mesenteric artery (arrow).

The patient reported that following her myocardial infarction, she began to experience severe aching midepigastric abdominal pain, progressing from predominately postprandial to occurring throughout the day. She reported chronic, unchanged alternating bowel habits of diarrhea and constipation, without signs of hematochezia or melena. A review of systems was notable for worsening nausea and abdominal bloating without weight loss. A physical exam was not performed due to the encounter being virtual. Laboratory values were notable for mild leukopenia (3.49 × 10^9 ^L^-1^), normocytic anemia (10.5 g/dL), and iron deficiency (ferritin 34.8 ng/mL), with normal electrolytes, renal function, lipase, and liver enzymes. The patient had undergone an upper endoscopy in the prior three months with documented features of gastric antral vascular ectasia, but no signs of peptic ulcer disease. Given the patient’s symptoms and history of atherosclerotic cardiovascular disease, a computed tomography angiogram was performed, which demonstrated moderate-to-severe stenosis of the proximal superior mesenteric artery (Figures 1-2), mild stenosis of the celiac trunk, and a patent inferior mesenteric artery without evidence of collateralization.

**Figure 3:**
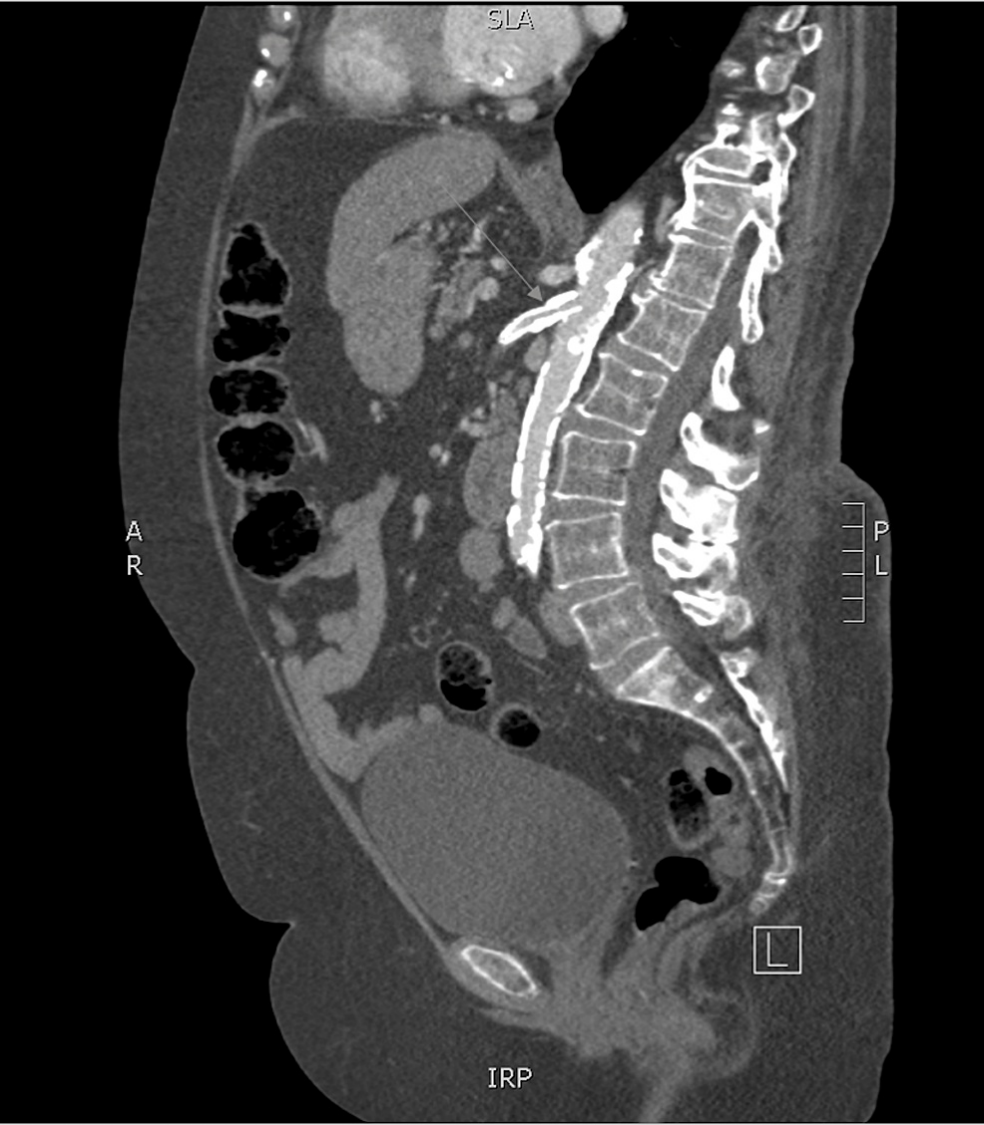
Sagittal CT of the abdomen and pelvis without contrast, demonstrating patency of the superior mesenteric artery after stent placement (arrow). CT, computed tomography

**Figure 4:**
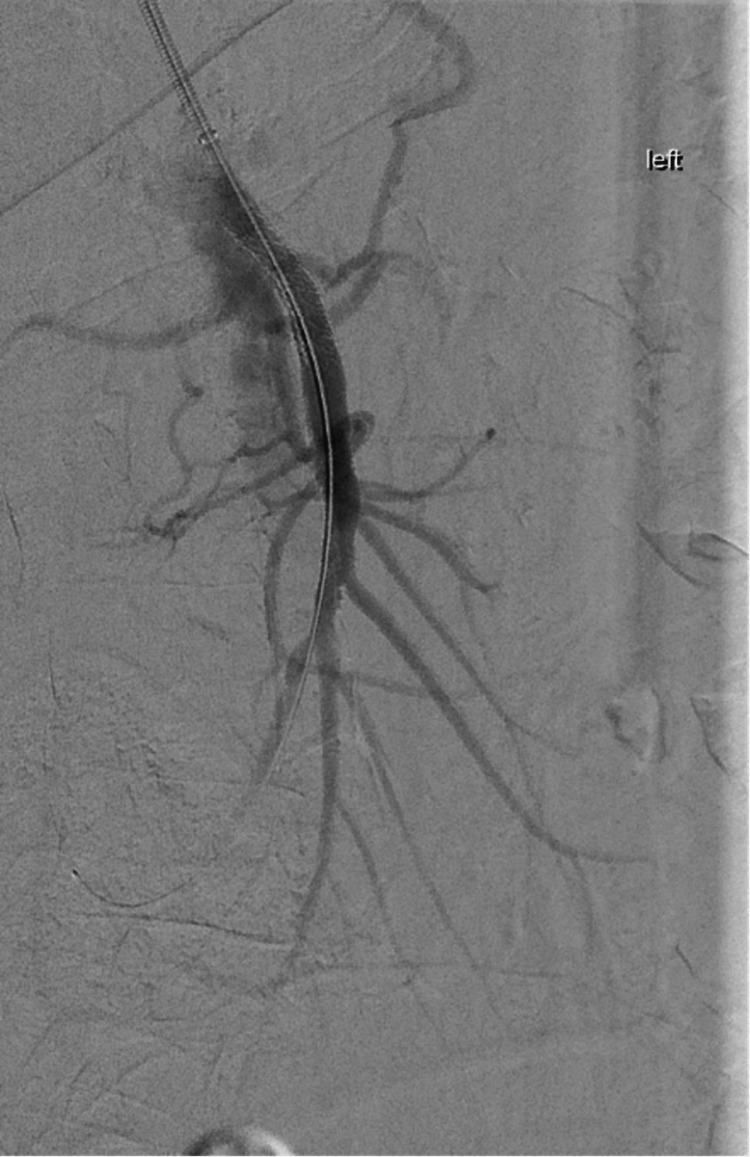
Mesenteric angiogram after the placement of the stent in the superior mesenteric artery, with patency and return of blood flow.

The patient was referred to vascular surgery, which performed successful endovascular stenting of the superior mesenteric artery with revascularization (Figures 3-4) with complete resolution of the patient’s abdominal pain.

## Discussion

Gastrointestinal tract involvement occurs almost universally in systemic sclerosis, with any area potentially affected [7-9]. While chronic mesenteric ischemia is not considered a typical manifestation, these patients are at an increased risk of ischemic atherosclerotic disease [5,10]. A recent meta-analysis of 31 articles by Au et al. concluded that patients with systemic sclerosis compared to healthy controls have an increased risk of developing clinical complications of atherosclerosis [5]. The studies in the meta-analysis evaluated cardiovascular disease with a variety of validated methods, including carotid intima-media thickness (IMT) and flow-mediated vasodilation (FMD, written as a percentage). Systemic sclerosis patients had an increased carotid IMT (summary mean difference 0.11 mm, 95% confidence interval (CI) 0.05-0.17 mm; *P* = 0.0006). Lower FMD measurements, which evaluate for endothelial dysfunction, are associated with an increase in cardiovascular events, whereas higher FMD% values are considered protective. Systemic sclerosis subjects had a lower FMD% compared to controls (summary mean difference -3.07%, 95% CI -5.44% to -0.69%; *P* = 0.01) [5]. In addition to chronic mesenteric ischemia, this patient was also affected by diffuse coronary artery disease at a young age; while limited systemic sclerosis is not her sole risk factor, it has likely contributed to her disease development.

Pathophysiologic causes contributing to atherosclerotic development for patients with systemic sclerosis are multifactorial, including systemic inflammation, altered lipid profiles, and endothelial dysfunction [5,8]. There are additional risk factors for chronic mesenteric ischemia due to the inherent vasculopathy of this condition, leading to the impaired ability to develop collateral vasculature [5,8]. The inability to form collaterals between mesenteric arteries leads to a greater likelihood of developing the symptomatic disease [1-2,4].

The relationship between systemic sclerosis and an increased risk for the development of chronic mesenteric ischemia is important to appreciate, given the high associated morbidity and mortality rates. Asymptomatic chronic mesenteric ischemia is associated with a five-year mortality of up to 40%, with most deaths attributed to a cardiovascular cause due to diffuse atherosclerotic disease; however, if all three main mesenteric arteries are affected, the five-year mortality increases up to 86% [11]. For those who develop acute mesenteric ischemia from mesenteric arterial thrombosis, mortality rates range from 50% to 70% [2]. Among patients with untreated symptomatic chronic mesenteric ischemia, the five-year mortality rate approaches 100%; thus, aggressive intervention is mandatory to prevent bowel infarction once symptoms develop [11-12].

All patients with established atherosclerotic disease should be managed with lifestyle inventions and medications to address modifiable risk factors such as hypertension and hyperlipidemia. Systemic sclerosis patients should be managed by a rheumatologist experienced in various organ-specific disease-modifying therapies. Vasodilator medications such as nitrates may provide temporary relief from the ischemic symptoms, but ultimately, symptomatic chronic mesenteric ischemia necessitates revascularization via either endovascular or open vascular surgery. Medical management and risk factor modification alone are reserved for patients deemed too high risk for either procedure or who have failed several previous revascularization attempts [13].

Several patient and treatment facility factors contribute to whether the open surgical or endovascular approach is chosen. Due to the higher initial success rate and a lower rate of perioperative complications, percutaneous endovascular intervention tends to be the preferred treatment method for chronic mesenteric ischemia and has a class IB recommendation from the American College of Cardiology/American Heart Association. Surgery is preferred for patients with a history of prior vascular complications, significant tortuosity or calcifications seen on imaging, or chronic mesenteric ischemia due to nonatherosclerotic causes or who have had a recurrence of stenosis despite multiple endovascular attempts at revascularization [13,14].

## Conclusions

Our case serves to improve awareness of the association between systemic sclerosis and the increased risk for atherosclerotic disease, including chronic mesenteric ischemia. Providers should follow this population closely with modifiable risk factors treated at an early stage and a low threshold to investigate for complications requiring aggressive revascularization.
